# The compatibility of diagnosis-intervention packet with the individualized treatment paradigm of traditional Chinese medicine: an analytical study

**DOI:** 10.3389/fpubh.2026.1851286

**Published:** 2026-06-26

**Authors:** Fan Ruoxi, Tu Lang, Li Shulei, Zhou Xiaoyuan

**Affiliations:** West China School of Public Health/West China Fourth Hospital, Sichuan University, Chengdu, China

**Keywords:** compatibility, diagnosis-intervention packet, interrupted time-series analysis, reform effectiveness, TCM

## Abstract

**Background:**

Traditional medicine (TM), including Traditional Chinese Medicine (TCM), serves as a vital component of global healthcare, yet lags behind modern medicine in standardized evaluation and payment model compatibility. In China, TCM's unique paradigm—centered on syndrome differentiation and non-surgical therapies—faces challenges under the Diagnosis-Intervention Packet (DIP) payment reform. However, systematic evidence on how DIP affects medical expenses across different levels of TCM service utilization remains scarce. This study aims to quantify DIP's effects on medical expenses between different groups, and to provide evidence for adjusting DIP policies based on TCM hospital characteristics.

**Method:**

We conducted a multiple-group interrupted time series analysis (MGITSA) on outcome variables reflecting hospitalization costs and TCM service structures, using a dataset containing 48,398 inpatient records from a TCM hospital in City C spanning from January 2018 to January 2021. The dataset included 4,343 records for the High-TCM group and 44,055 records for the Low-TCM group.

**Results:**

MGITSA indicated that after DIP implementation, the High-TCM group experienced significant increases in both hospitalization costs (*p* < 0.01) and out-of-pocket payments (*p* < 0.05). Conversely, the low-TCM group experienced significant decrease in hospitalization cost (*p* < 0.01) and Chinese herbal decoction pieces (*p* < 0.01).

**Conclusion:**

DIP exerts distinct short-term effects: the high-TCM group maintained cost stability, while the low-TCM group exhibited cost inflation. Policy adjustments, such as differential DIP scoring based on TCM treatment rates, are needed to enhance compatibility and support TCM's sustainable development.

## Background

Traditional medicine (TM), encompassing systems such as Traditional Chinese Medicine (TCM), Ayurveda, Unani, and European herbal medicines, holds profound historical and cultural significance as a vital component of global healthcare (1). Nowadays, TM still remains crucial, especially in developing countries and rural areas, due to its accessibility, affordability, and alignment with local cultural values (2). According to the World Health Organization (WHO), about 80% of the population in Africa and Asia depends on traditional medicine for primary health care (3).

However, TM continues to lag behind modern medicine (MM) in global development. It is often perceived as pre-scientific, with its methods and remedies viewed as candidates for replacement by modern, more effective, science-based medical approaches (4). Over the past decades, efforts to promote the safety, efficacy and quality of TM have involved navigating a trade-off between the benefits of western medicine and those of TM itself. In China, for example, traditional Chinese medicine (TCM) has evolved into a hybrid mode, particularly in TCM hospitals and TCM departments in general hospitals. These institutions increasingly integrate clinical diagnostics, examination techniques, and pharmaceutical interventions from Western medicine, while continuing to provide traditional treatments such as acupuncture and herbal therapies to patients in need. The proportions of traditional medicine vs. modern interventions vary depending on different diagnosis and clinic symptoms.

Globally, health systems are placing growing emphasis on traditional medicine. The WHO Traditional Medicine Strategy (2014–2023) recognized the role of traditional medicine in primary care and preventive health, and advocated for standardized regulation and enhanced research to ensure its safety, efficacy, and equitable access (5). Some traditional treatment modalities, due to their demonstrated effectiveness and low cost, have gradually been incorporated into national health financing systems (5). For instance, Kim et al. (6), after investigating the acceptance of different payment models among Korean doctors, found that a mixed payment model of per-visit and episode-based model was perceived as appropriate for traditional Korean medicine providers. Ros et al. (7) explored the inclusion of traditional medicine in Cambodia's health insurance system as a means of reducing the financial burden on low-income families. Lee et al. (8) compared outpatient costs between traditional Korean medicine and Western medicine for lumbar disc herniation to assess the feasibility of including TM in existing health insurance payment schemes. Suharmiati et al. (9) observed that a growing demand for TM in Indonesia's community medical services, highlighting the urgency of incorporating TM in national health insurance payment systems.

Against this backdrop, the redesign of provider-payment mechanisms has become a key policy lever for improving technical and allocative efficiency in social health insurance. The Diagnosis-Intervention Packet (DIP) payment system is a novel medical insurance payment method in China (10). It aims to optimize the allocation of medical resources, enhance utilization efficiency, promote the decentralization of medical service, alleviate hospital pressure, contain the growth of medical costs, and strengthen quality supervision and incentive mechanisms to improve medical service quality (11). However, TCM hospitals encounter structural disincentives under the DIP payment system reform. Their case-mix is characterized by (i) a high share of TCM-specific interventions, (ii) reliance on syndrome-differentiation diagnostics that are not codable within the ICD-10 taxonomy underpinning the current grouping algorithm, and (iii) a preventive-rehabilitative care philosophy that lengthens average inpatient stay (12, 13). These features compress the effective price per case, reduce measured efficiency, and risk downward pressure on reimbursements, thereby undermining the sector's financial sustainability and potentially eroding the supply of TCM services.

Prior analyses have signaled ICD-10 grouping misalignment and depressed TCM-specific valuation weights under the DIP, yet no study has quantified the policy's causal effect on cost containment or on the composition of TCM vs. non-TCM spending within TCM hospitals. This evaluation fills that evidence gap by measuring the impact of a locally-adapted DIP bundle on total episode costs and on the TCM cost share of an acute-care TCM hospital in City C. Findings will inform actuarial recalibration of TCM group weights and guide national guidelines that safeguard financial protection while preserving TCM service supply.

## Methods

### Study design

On July 1st, 2020, the adjusted-DIP payment method was implemented in City C in China, replacing the previous fee-for-service payment model. We adopted the multiple-group interrupted time series (MGITSA) design to assess the impact of this reform on inpatient services in TCM hospitals. The study period was from January 1st, 2018, to January 31st, 2021, with July 1st, 2020, as the intervention point. The pre-intervention stage was from January 2018 to June 2020, and the post-intervention stage was from July 2020 to January 2021.

To explore the impact of payment reform on TCM more accurately, we grouped hospitalization cases in TCM hospitals according to TCM quota. Referring to the definition of the “TCM Treatment Rate” used in City Z (the proportion of costs for Chinese herbal decoction pieces, TCM service items, and Chinese patent medicine among total hospitalization costs) (14), we applied this concept to our dataset and examined the distribution of the calculated TCM cost proportion across our patient cohort. The number of cases sharply decreased from 4,465 in the 40%−50% TCM cost proportion bin to 2,780 in the 50%−60% bin, with further substantial declines in higher bins (1,318 in 60%−70%, 241 in 70%−80%). Therefore, we pragmatically set the threshold for grouping patients: patients whose TCM treatment rate is not less than 50% are classified as high-TCM group, while the others are categorized as low-TCM group. We compared the changes in hospitalization costs and TCM service items costs before and after the DIP implementation and evaluated the differences in effects between the two groups. Multiple-group interrupted time series (MGITSA) analysis facilitates us to make causal inference.

### Data source

Our study sampled a TCM hospital in City C, which started DIP payment settlement on July 1st, 2020, as a designated DIP medical insurance payment institution in City C. The data were obtained from the Hospital Information System (HIS), including 55,158 inpatient cases of basic medical insurance from January 2018 to January 2021. All data were anonymized to protect the privacy of individuals. After excluding cases with length of stay >60 days (due to exemption from DIP payment) or logical errors, 48,398 cases were eligible for our study. The dataset included patients' demographic information, length of stay, hospitalization costs, and TCM service items costs, etc. The costs were adjusted to the price in 2026 and converted to United States Dollars (USD) using the exchange rate of 1 Chinese Yuan (CNY) ≈ 0.15 USD as of May 9, 2026 (Table 1).

**Table 1:** Variable definitions.

Variable type	Variables	Definition/measurement
Grouping variable	TCM treatment rate	Proportion of costs for Chinese herbal decoction pieces, TCM service items, and Chinese patent medicine in total hospitalization costs
	High-TCM group	Patients with TCM treatment rate ≥50%.
	Low-TCM group	Patients with TCM treatment rate < 50%
Outcome variables	Average hospitalization cost per case	Total hospitalization expenses (USD) per case.
	Average out-of-pocket (OOP) payment per case	Patient's self-paid expenses (USD) excluding insurance coverage.
	Average cost of Chinese herbal decoction pieces per case	Expenses for Chinese herbal decoction pieces (USD) per case.
	Average cost of Chinese patent medicine per case	Expenses for Chinese patent medicine (USD) per case.
	Average cost of TCM service items per case	Expenses for TCM services (USD) per case.
	Average cost of Western pharmaceuticals per case	Expenses for Western drugs (USD) per case.

### Propensity score matching (PSM)

To minimize the impact of individual differences on the results and to address the imbalance caused by the significant difference in the size of the two groups, Stata 16.0 was used to perform one-to-one nearest neighbor matching with propensity scores for patients treated primarily by Chinese medicine and those treated with a combination of Chinese and Western medicine. We employed a logistic regression analysis model with cost indicators as dependent variables and patient gender, age, length of stay (log) and ICD-10 codes as independent variables. The selected variables were identified through a literature review (15). The ICD-10 codes were grouped into three clinically meaningful categories: Internal Medicine (IM), Surgery (Surg), and else. The grouping logic was informed by the patient classification approach of DIP payment system, which groups patients according to the combination of principal diagnosis ICD-10 codes and procedure ICD-9-CM-3 codes (16, 17). Specifically, Surg group was assigned to cases whose primary diagnosis fell under ICD-10 chapters S–T (injury, poisoning) or where a procedure code indicated operative intervention; the IM group comprised diagnoses from chapters A–R typically not requiring operative management; and the else group, containing mainly chapter Z codes (factors influencing health status) and uncategorizable low-volume diagnoses, served as the reference category. The propensity score for each patient was calculated using the regression equation, with a threshold value of 0.05.

### Multiple-group interrupted time series analysis (MGITSA)

The MGITSA is a quasi-experimental design that effectively evaluates the impact of policy reforms. When other external factors change slowly over a short period, this method can effectively assess the outcomes of policy reforms. It has been widely used to evaluate the effectiveness of public health policies and interventions (18, 19).

We used a generalized linear segmented regression model to evaluate the impacts of the DIP payment reform on six key indicators—average hospitalization expense per case, average OOP payment per case, average TCM-related expenses per case, and average cost of western pharmaceuticals per case, before and after the implementation of the reform. We estimated the model using Driscoll–Kraay standard errors (20) with a lag order of 1, which was selected based on the autocorrelation function (ACF) and partial autocorrelation function (PACF) of the residuals as well as the Ljung–Box *Q* test (all indicating no significant autocorrelation). Additionally, we conduct a horizontal comparison to examine the differences in the trends of these six indicators across different groups (21). Seasonality was examined by including month-of-year dummies; the joint test was not significant for most outcomes, and sensitivity analyses confirmed that adjusting for seasonality did not alter the main conclusions. According to the patients' discharge dates, Stata 16.0 was used to mark observations monthly (37 months in total) and perform all statistical analyses. The segmented regression model is shown below:


$$\begin{array}{r} Y_{\text{t}}=\beta_{0}+\beta_{\text{1}}T_{\text{t}}+\beta_{\text{2}}X_{\text{t}}+\beta_{\text{3}}X_{\text{t}}T_{\text{t}}+\beta_{\text{4}}Z+\beta_{\text{5}}ZT_{\text{t}}\\ +\beta_{\text{6}}ZX_{\text{t}}+\beta_{\text{7}}ZX_{\text{t}}T_{\text{t}}+\varepsilon_{t} \end{array}$$

Here *Y*_t_ represents the outcome variable, which includes costs for hospitalization, Chinese herbal decoction pieces, TCM service items, Chinese patent medicine, Western pharmaceuticals and OOP payment; *Z* is a group dummy variable (*Z* = 1 for the high-TCM group, *Z* = 0 for the Low-TCM group); β_0_ is the constant term, representing the initial level of Y in the Low-TCM group; β_1_ represents the slope of the Low-TCM group before the reform; β_2_ indicates the change in the level of the low-TCM group during the reform; β_3_ is the difference in slope of the Low-TCM group before and after the reform; β_4_ represents the level difference between groups before the reform; β_5_ represents the difference in slope between groups before the reform; β_6_ indicates the change in level between groups during the reform; β_7_ reflects the difference in slope change between groups after the reform; β_8_ (β_8_ = β_1_ + β_3_) reflects the short-term trend after the intervention; β_9_ (β_9_ = β_1_ + β_3_ + β_5_ + β_7_) measures the short-term trend of the high-TCM group after the reform; ε_*t*_ represents random error. This study uses July 2020 as the policy intervention time point for the DIP payment method reform.

## Results

### Sample characteristics

Table 2 presents the sample characteristics for the High-TCM group and low-TCM group, respectively. The table showed significant differences in gender, age, length of stay and ICD-10 codes between groups. The high-TCM group (*n* = 4,343) included 38.89% males and 61.11% females; < 50 years, 50–69 years, and ≥70 years accounted for 39.53%, 46.03%, and 14.44%, respectively; length of stay (log < 2.5 vs. ≥2.5) accounted for 39.26% and 60.74%. The Low-TCM group (*n* = 44,055) included 44.74% males and 55.26%; < 50 years, 50–69 years, and ≥70 years accounted for 36.49%, 36.57%, and 26.94%, respectively; length of stay (log < 2.5 vs. ≥2.5) accounted for 74.91% and 25.09%. Regarding ICD-10 classification, the high-TCM group was heavily concentrated in internal medicine diagnoses (55.01%), with a substantial proportion of surgical diagnoses (44.41%), whereas the low-TCM group exhibited a predominance of internal medicine cases (76.11%) and a smaller share of surgical cases (21.88%). Other diagnoses accounted for less than 2% in both groups.

**Table 2:** Description of univariate analysis of two groups.

Variables	High-TCM group (*n* = 4,343)	Low-TCM group (*n* = 44,055)	*X*^2^/*Z*	*P*-value
Gender	
Male	1,689 (38.89%)	19,711 (44.74%)	54.88	< 0.001
Female	2,654 (61.11%)	24,344 (55.26%)		
Age (years)	
< 50	1,717 (39.53%)	16,076 (36.49%)	343.93	< 0.001
50–69	1,999 (46.03%)	16,110 (36.57%)		
≥70	627 (14.44%)	11,869 (26.94%)		
Length of stay (days)	
< 2.5 (log)	1,705 (39.26%)	33,003 (74.91%)	2.5 × 10^3^	< 0.001
≥2.5 (log)	2,638 (60.74%)	11,052 (25.09%)		
ICD-10 classification	
Internal medicine (IM)	950 (55.01%)	29,652 (76.11%)	482.78	< 0.001
Surgery (Surg)	767 (44.41%)	8,526 (21.88%)		
Else	10 (0.58%)	784 (2.01%)		

### PSM result

PSM 1:1 nearest neighbor matching showed large pre-matching covariate distribution deviations, which were reduced to ≤ 10% after matching (Table 3).

**Table 3:** PSM matching results.

Variables	Mean		Standardization bias/%	Standard deviation changes/%	*t* value	*P* value
	High-TCM group	Low-TCM group				
Gender	
Pre-matching	0.46	0.47	−0.7	−2.6	−0.20	0.838
Post-matching	0.47	0.36	0.7		0.21	0.836
Age	
Pre-matching	52.85	53.27	−2.6	23.2	−0.77	0.442
Post-matching	53.38	53.05	2.0		0.59	0.555
Length of stay (days)	
Pre-matching	2.27	2.21	6.1	88.9	1.80	0.072
Post-matching	2.25	2.25	0.7		0.20	0.842
IM	
Pre-matching	0.55	0.53	3.6	96.7	1.06	0.290
Post-matching	0.55	0.55	0.1		0.03	0.972
Surg	
Pre-matching	0.44	0.46	−4.0	81.9	−1.16	0.245
Post-matching	0.45	0.45	0.7		0.21	0.836

### The results of multi-group interrupted time series analysis

**Figure 1:**
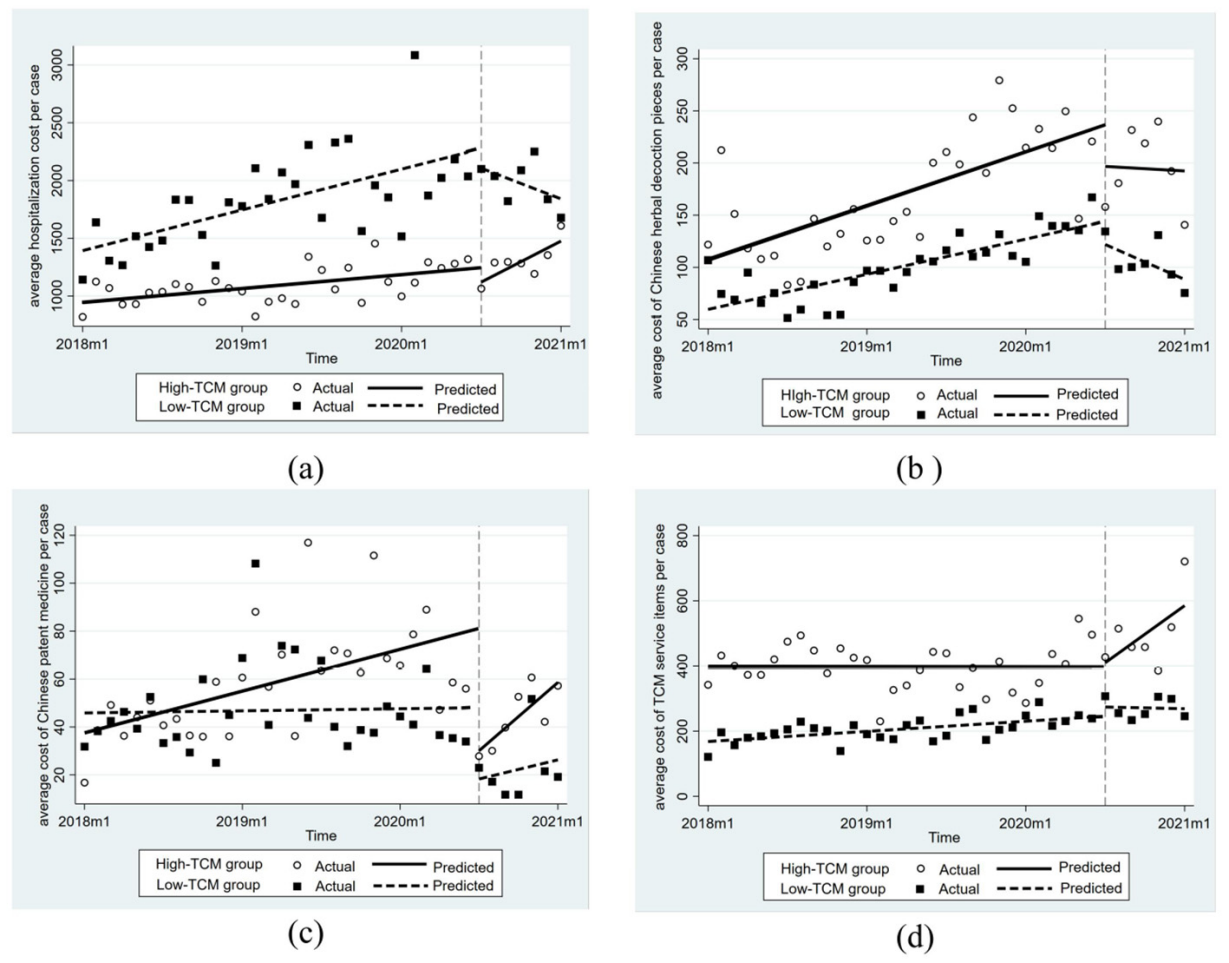
Trends of the average hospitalization cost per case before and after DIP reform **(a)**, trends of the average cost of Chinese herbal decoction pieces per case before and after DIP reform **(b)**, trends of the average cost of Chinese patent medicine per case before and after DIP reform **(c)**, trends of the average cost of TCM service items per case before and after DIP reform **(d)**.

After DIP implementation, the Low-TCM group showed significant decrease in two key cost indicators, including average hospitalization cost per case (β_3_ = −73.61, *p* < 0.01) and the average cost of Chinese herbal decoction pieces (β_3_ = −8.40, *p* < 0.01); the post-reform short-term trend for herbal decoction pieces was also significant (β_8_ = −5.59, *p* < 0.05). While other TCM cost indicators did not change significantly (all *p* > 0.05). Between-group comparisons showed that the high-TCM group experienced a significantly steeper increase in hospitalization cost slope (β_7_ = 122.70, *p* < 0.01), leading to a positive short-term trend for the high-TCM group (β_9_ = 59.15, *p* < 0.01). For Chinese patent medicine, the high-TCM group also had a significantly steeper increase (β_7_ = 2.05, *p* < 0.05), with a positive post-reform trend (β_9_ = 4.76, *p* < 0.001). No significant between-group differences were found for other cost indicators (all *p* > 0.05; Table 4 and Figure 1).

**Table 4:** Analysis results of changes in medical expenses of different groups in TCM hospital in the double-group intermittent time series model.

Index	Average hospitalization cost per case (USD)	Average out-of-pocket payment per case (USD)	Average cost of Chinese herbal decoction pieces per case (USD)	Average cost of Chinese patent medicine per case (USD)	Average cost of TCM service items per case (USD)	Average cost of western pharmaceuticals per case (USD)
β_0_	1363.74 (17.42)^***^	778.44 (10.27)^***^	56.86 (5.56)^***^	45.79 (8.07)^***^	165.52 (17.06)^***^	262.62 (9.75)^***^
β_1_	29.38 (5.27)^***^	14.39 (3.34)^**^	2.81 (5.59)^***^	0.07 (0.27)	2.60 (4.71)^***^	5.95 (2.81)^**^
β_2_	2113.77 (2.63)^*^	524.84 (0.65)	238.26 (2.78)^**^	−68.82 (−1.41)	135.45 (0.77)	480.35 (2.08)^*^
β_3_	−73.61 (−2.92)^**^	−16.81 (−0.69)	−8.40 (−3.22)^**^	1.26 (0.84)	−3.47 (−0.68)	−19.17 (−2.83)^**^
β_4_	−432.06 (−4.94)^***^	−92.45 (−1.45)	45.45 (2.75)^**^	−9.57 (−2.22)^*^	229.84 (9.95)^***^	−188.12 (−7.27)^***^
β_5_	−19.32 (−3.11)^**^	−14.12 (−3.19)^**^	1.51 (1.45)	1.38 (4.66)^***^	−2.58 (−1.37)	−4.57 (−2.14)^*^
β_6_	−3758.20 (−2.76)^**^	−2245.94 (−1.99)	−121.38 (−0.46)	−84.87 (−1.73)	−1025.79 (−1.59)	−554.82 (−2.04)^*^
β_7_	122.70 (2.92)^**^	69.79 (2.00)^*^	3.35 (0.44)	2.05 (1.38)	32.64 (1.65)	20.55 (2.60)^**^
β_8_	−44.23 (−1.80)	−2.42 (−0.10)	−5.59 (−2.23)^*^	1.33 (0.90)	−0.87 (−0.17)	−13.21 (−1.97)
β_9_	59.15 (3.25)^**^	53.26 (2.65)^*^	−0.72 (−0.09)	4.76 (4.65)^***^	29.19 (1.70)	2.75 (1.42)

^*^p < 0.05, ^**^p < 0.01, ^***^p < 0.001; the t value of statistical test is in parentheses.

**Figure 2:**
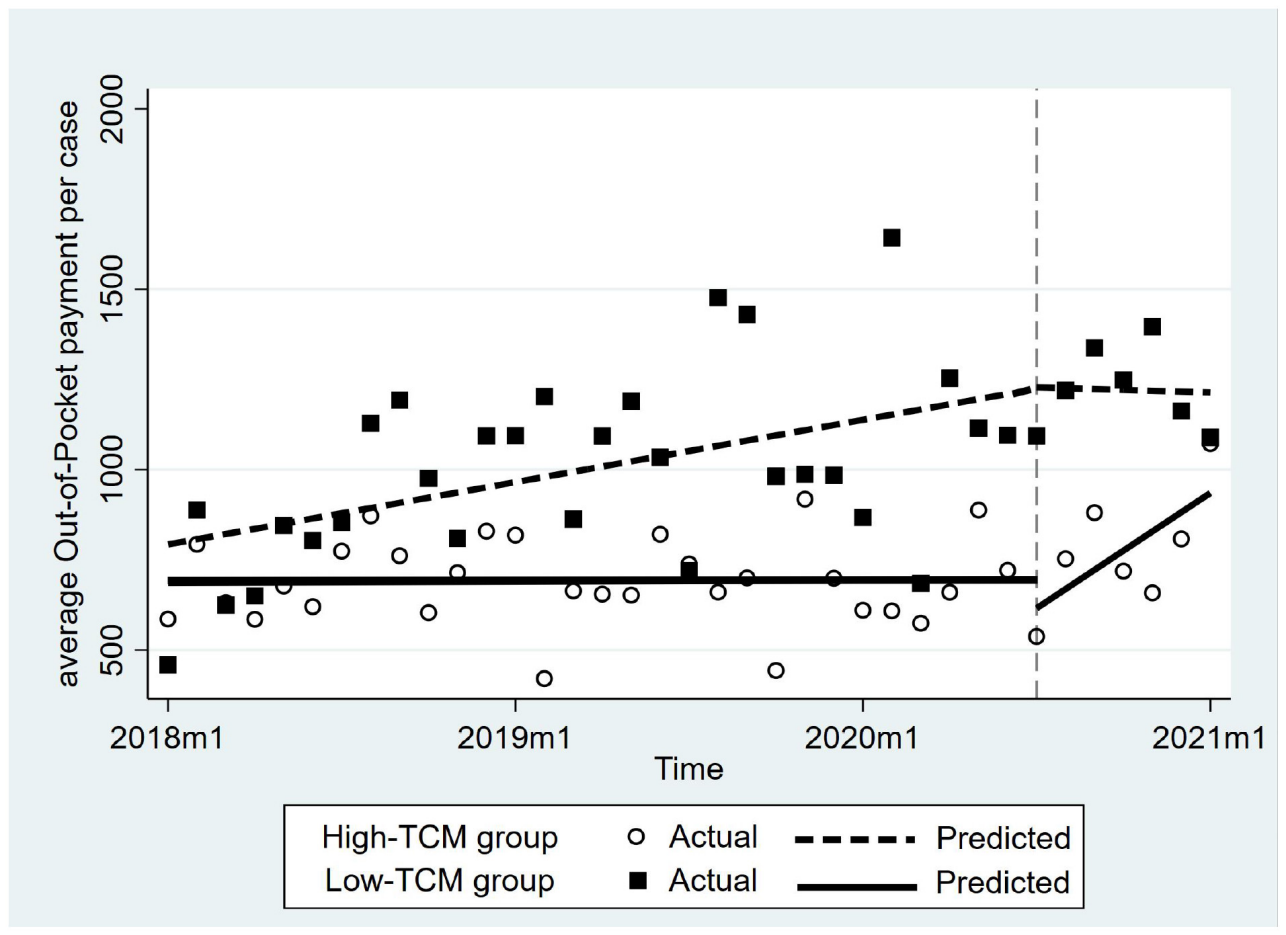
Trends of the average out-of-pocket payment per case before and after DIP reform.

In the low-TCM group, the slope of average OOP payment per case did not change significantly after the reform (β_3_ = −16.81, *p* = 0.49), and its post-reform short-term trend was also not significant (β_8_ = −2.42, *p* = 0.92). However, the between-group difference in slope change was significant (β_7_ = 69.79, *p* < 0.05), indicating that the high-TCM group experienced a greater increase in OOP slope after the reform. And the short-term trend for the high-TCM group was a significant increase (β_9_ = 53.26, *p* < 0.05; Table 4 and Figure 2).

**Figure 3:**
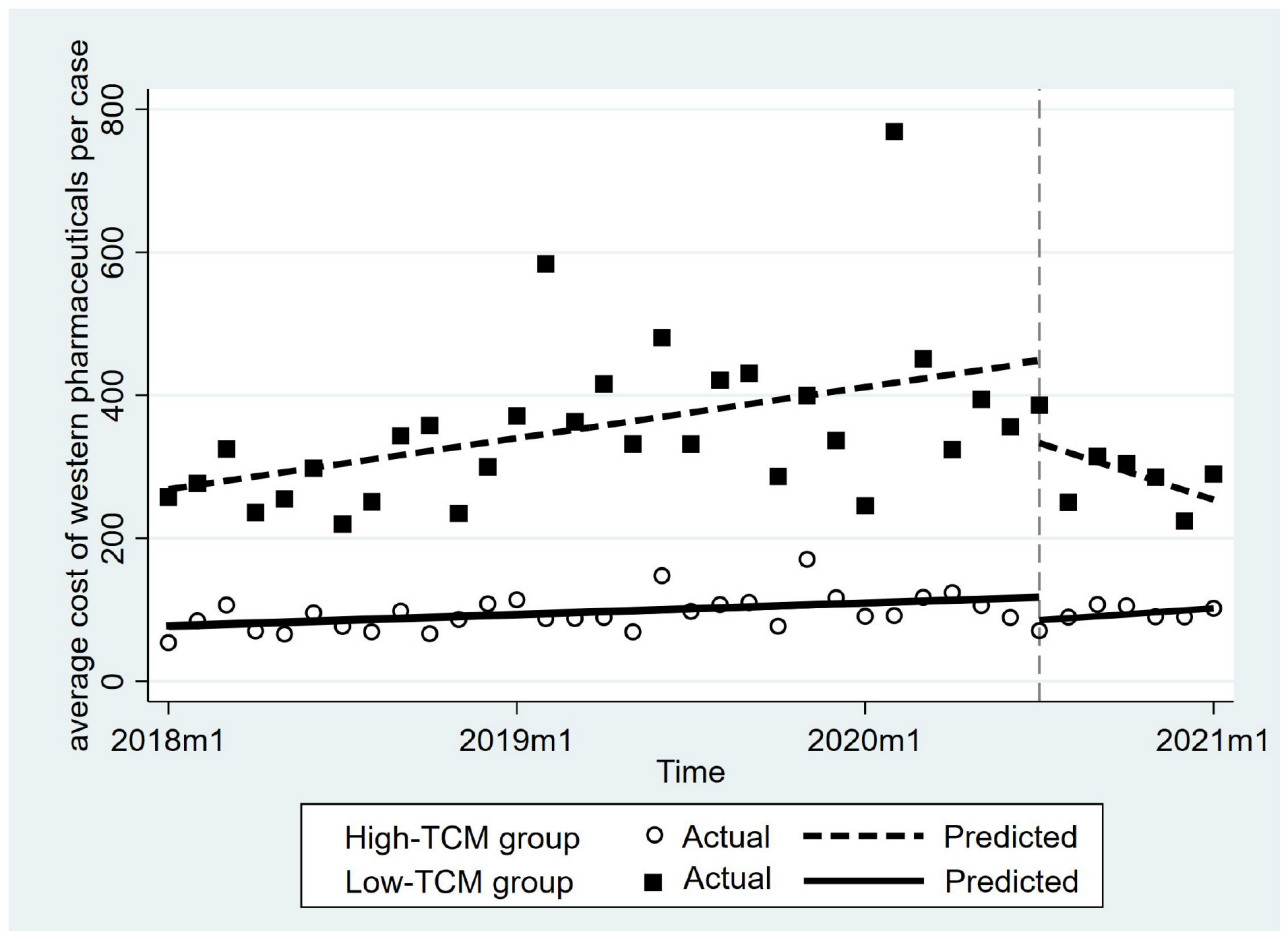
Trends of the average cost of western pharmaceuticals per case before and after DIP reform.

In the Low-TCM group, the slope of average cost of western pharmaceuticals decreased significantly after the reform (β_3_ =−19.17, *p* < 0.01), but the post-reform short-term trend was not significant (β_8_ = −13.21, *p* = 0.056). The between-group difference in slope change was significant (β_7_ = 20.55, *p* < 0.01), indicating a less steep decline in the High-TCM group. However, the short-term trend was not significant (β_9_ = 2.75, *p* = 0.16; Table 4 and Figure 3).

### Robustness test

To verify the robustness of the research findings, this study adjusted the implementation date of the DIP policy to January 1, 2020. After adjusting the policy implementation date, the changes and mechanisms of the cost indicators were highly consistent with the original study results, without any contradictions arising from the change in the timing of the policy. This indicates that the impact of the DIP payment reform on the costs of different treatment groups in TCM hospitals is inherently stable, and the research conclusions are not significantly affected by the choice of the policy implementation date, thus verifying the robustness of the findings (Table 5).

**Table 5:** Robustness test results.

Index	Average hospitalization cost per case (USD)	Average out-of-pocket payment per case (USD)	Average cost of Chinese herbal decoction pieces per case (USD)	Average cost of Chinese patent medicine per case (USD)	Average cost of TCM service items per case (USD)	Average cost of western pharmaceuticals per case (USD)
β_0_	1319.97 (15.69)^***^	729.62 (8.85)^***^	61.61 (5.74)^***^	41.47 (8.57)^***^	170.23 (14.47)^***^	255.39 (11.96)^***^
β_1_	33.94 (4.43)^***^	19.58 (2.88)^**^	2.32 (3.67)^**^	0.52 (1.20)	2.05 (2.23)^*^	6.59 (4.07)^***^
β_2_	1447.74 (1.69)	43.27 (0.10)	185.60 (3.35)^**^	64.76 (2.47)^*^	12.83 (0.20)	684.03 (2.19)^*^
β_3_	−57.39 (−2.03)^*^	−6.79 (−0.44)	−6.40 (−3.49)^**^	−2.93 (−3.00)^**^	0.40 (0.18)	−25.50 (−2.63)^*^
β_4_	−379.36 (−4.35)^***^	−47.52 (−0.66)	32.93 (1.58)	−13.79 (−2.51)^*^	251.13 (14.43)^***^	−187.66 (−9.05)^***^
β_5_	−24.59 (−2.82)^**^	−18.67 (−2.46)^*^	2.78 (1.97)	1.81 (3.43)^**^	−4.59 (−3.29)^**^	−4.49 (−2.56)^*^
β_6_	−1911.52 (−2.06)^*^	−727.51 (−1.24)	9.22 (0.13)	28.88 (1.13)	−527.02 (−1.96)	−636.21 (−2.05)^*^
β_7_	73.19 (2.34)^*^	29.49 (1.40)	−1.49 (−0.56)	−1.57 (−1.63)	20.01 (2.19)^*^	22.83 (2.38)^*^
β_8_	−23.45 (−0.91)	12.79 (0.34)	−4.07 (−2.41)^*^	−2.41 (−2.76)^**^	2.46 (1.21)	−18.91 (−2.05)
β_9_	25.15 (2.52)^*^	23.61 (2.66)^*^	−2.78 (−1.20)	−2.16 (−2.02)	17.88 (2.40)	−0.56 (−0.60)

^*^P < 0.05, ^**^P < 0.01, ^***^P < 0.001; the t value of statistical test is in parentheses.

## Discussion

Multiple-group interrupted time series (ITSA) on 48,398 inpatient records from a tertiary TCM hospital in City C (2018–2021) showed that the Diagnosis-Intervention Packet (DIP) reform generated divergent cost trajectories depending on the TCM-treatment share of the case-mix. Our MGITSA analysis reveals three notable findings:

i) In the high-TCM group, the short-term trend of average hospitalization cost per case showed a significant increase after the reform (β_9_ = 59.15, *p* < 0.01). Similarly, the short-term trend of average OOP payment also increased significantly (β_9_ = 53.26, *p* < 0.05). These findings suggest that, contrary to the expectation that TCM-dominant care would be financially protected under DIP, patients in this group actually experienced higher total hospitalization costs and greater OOP burden in the immediate post-reform period. The results appear to differ from findings reported in City Z, where DIP significantly reduced total costs and OOP payments for TCM-characterized diseases (22). Two factors may explain this discrepancy. First, our study captured the full hospital-wide inpatient case-mix, including non-advantageous and mixed-treatment cases. Second, City Z's DIP framework had undergone multiple rounds of score calibration, while City C was still in the early implementation phase in our research. Long-term monitoring is needed to assess whether these upward trends persist or reverse as the DIP framework matures.
ii) In stark contrast, within the Low-TCM group (< 50% TCM costs), the average hospitalization cost per case changed significantly after the reform (β_3_ = −73.61, *p* < 0.01), but the post-reform short-term trend was not significant (β_8_ = −44.23, *p* = 0.071), meaning that a sustained reduction in total hospitalization costs was not achieved within the 7-month observation. The average OOP payment did not change significantly in either slope (β_3_ = −16.81, *p* = 0.490) or short-term trend (β_8_ = −2.42, *p* = 0.920). This phenomenon differs from the classical cost-transfer hypothesis, which predicts that hospitals facing financial pressure under fixed payments may shift costs to patients through higher OOP charges (23). One plausible explanation is that cases with lower TCM intensity tend to follow more standardized diagnostic and treatment protocols, which are easier to align with DIP's grouping logic. Consequently, hospitals could absorb cost savings without transferring financial pressure to patients, at least in the immediate post-reform period. However, the lack of a significant sustained trend suggests that the cost-containment effect may be temporary or that the observation period was too short to capture full behavioral adjustments. Longer follow-up and further verification are needed to determine whether the observed slope changes in the low-TCM group will evolve into stable long-term cost savings and whether any latent cost-shifting emerges over time.
iii) The divergent shifts in TCM service costs between the groups further illuminate hospital strategic adaptations. The “counter-trend growth” of Chinese patent medicine in the High-TCM group (β_9_ = 4.76, *p* < 0.001) highlights its role as a standardized, DIP-friendly TCM modality. Unlike customized herbal decoctions, patent medicines have fixed costs and dosages, making them easier to integrate into standardized clinical pathways and cost accounting under DIP, akin to Western pharmaceuticals (24). Conversely, the lack of a similar increase within the Low-TCM group suggests that hospitals reserved this “DIP-friendly” strategy predominantly for cases already committed to a TCM-dominant treatment pathway. The most notable change in the Low-TCM group was a significant decrease in the average cost of Chinese herbal decoction pieces (β_3_ = −8.40, *p* < 0.01), with a sustained short-term downward trend (β_8_ = −5.59, *p* < 0.05). This decline can be interpreted as the fixed payment effectively curbed the use of cost-variable, individualized herbal prescriptions that lack standardized pricing. From a cost-containment perspective, this represents a successful alignment of provider incentives with payment rules (25). However, the reduction in customized decoctions—while financially beneficial—warrants ongoing monitoring to ensure that cost control does not inadvertently erode the core TCM practice of syndrome differentiation and personalized herbal treatment.

At the same time, in the low-TCM group, the average cost of Western pharmaceuticals showed a significant decrease in slope after the reform (β_3_ = −19.17, *p* < 0.01), indicating that DIP successfully curbed Western drug spending in cases with lower TCM intensity. However, the post-reform short-term trend for this group was not significant (β_8_ = −13.21, *p* > 0.05), suggesting that the decline was not sustained as a stable trend within the 7-month observation. Between-group comparisons revealed a significant difference in slope (β_7_ = 20.55, *p* < 0.01), meaning that the high-TCM group had a relatively less steep decline. Nevertheless, the short-term trend for the high-TCM group was not significant (β_9_ = 2.75, *p* > 0.05). This finding differs from previous studies that reported a persistent reliance on Western treatment pathways in TCM hospitals (26). One possible explanation is that the DIP reform's cost-control pressure was absorbed through other channels rather than through adjustments in the use of Western pharmaceuticals. Alternatively, the 7-month post-reform observation period may be too short to capture shifts in prescribing behavior. Further monitoring over a longer timeframe is needed to determine whether DIP ultimately alters the use of Western drugs in TCM settings (27).

## Conclusions

In conclusion, DIP appears not compatible with TCM-dominant care, and policy adjustments—such as differential scoring based on TCM treatment rates—are needed to enhance this compatibility and support the sustainable development of TCM. Adjusting group weights by TCM-treatment intensity and expanding the classification taxonomy are necessary design corrections to align provider incentives with the policy objective of sustaining equitable, high-quality traditional medicine services within universal health coverage.

Future research should focus on exploring dynamic DIP score adjustments based on the “TCM treatment rate” (28). In top-level design, it is necessary to ensure that the reform of medical insurance payment systems considers the compatibility between TM and the Western medicine system from the source.

Meanwhile, given the study's findings on cost-stabilizing effects of standardized TCM services and pathway dependency in TCM-MM treatments, continuous monitoring of DIP implementation is essential (29). Only in this way can DIP truly transform from a cost-control tool into a catalyst for enabling the inheritance and innovation of traditional medicine in the modern healthcare system.

## Limitations

This study has several limitations that need to be addressed: first, single-center data limits generalizability, but our findings signal universal tensions between standardization and individualized TM paradigms warranting multi-region studies. Second, the observation period after DIP reform is only 7 months, and long-term effects need to be tracked later. Third, although we adjusted for ICD-10 categories in the propensity score model, we lacked direct measures of disease severity, which may have residual confounding. Finally, regional differences in the threshold of TCM treatment rate may affect the external validity of the study. Longitudinal multi-center studies—coupled with adjustments to the TCM treatment rate threshold across regions—are crucial to validate these results and refine DIP's compatibility with TCM practice.

## Data Availability

The original contributions presented in the study are included in the article/supplementary material, further inquiries can be directed to the corresponding author.
